# Inhibition of HMGB1 Might Enhance the Protective Effect of Taxifolin in Cardiomyocytes via PI3K/AKT Signaling Pathway

**DOI:** 10.22037/ijpr.2020.113584.14384

**Published:** 2021

**Authors:** Erjun Feng, Jian Wang, Xinwei Wang, Zhenguo Wang, Xiaochu Chen, Xu Zhu, Wenli Hou

**Affiliations:** a *Department of Cardiology, Fourth Center Hospital of Tianjin, Tianjin, China, 300000. *; b *Oncology Department of Characteristic Medical Center of PAF, Tianjin, China, 300162. *; c *Medical Research Department of Characteristic Medical Center of PAF, Tianjin, China, 300162. *; d *Second Department of Neurology, Central Hospital of Handan, Handan, China, 056000. *; e *Cadre Ward of Characteristic Medical Center of PAF, Tianjin, China, 300162.*; 1 *E. F. and J. W. and X. W. contributed equally to this work.*

**Keywords:** HMGB1, Taxifolin, Cardiomyocytes, PI3K/AKT signaling pathway

## Abstract

Cardiovascular diseases (CVD) affect millions of people and spend a lot of medical costs around the world each year. Taxifolin is a natural anti-oxidative reagent obtained from multiple plants and exhibits a wide range of pharmacological effects. High mobility group box protein 1 (*HMGB1*) is expressed in multiple types of cells in the extracellular environment, regulating the pro-inflammatory process. Here, we detected the viability of cells using MTT assay, and the expression of each target protein was detected using western blotting analysis. The expression of each target mRNA was detected using the qPCR method, and the concentration of each cytokine in serum samples was detected using the ELISA method. In this study, we found that taxifolin could decrease the expression of hypoxia-inducible factor-1α (HIF-1α) while increasing the expression of endothelial nitric oxide synthase (eNOS), presented a protective role. Besides, taxifolin could also increase the expression of vascular endothelial growth factor-α (VEGF-α), transforming growth factor-β (TGF-β) and fibroblast growth factor21 (FGF21), resulting in viability rate increasing. And these effects were mediated by phosphatidylinositol 3-hydroxy kinase (PI3K)/AKT/mTOR signaling pathway; a similar trend was also observed in *HMGB1* knockdown mice. We also found that inhibition of *HMGB1* could enhance the cardioprotective effect of taxifolin and might be a new therapeutic strategy for cardiovascular disease.

## Introduction

Cardiovascular disease (CVD) is a series of diseases related to heart or blood vessels, including heart failure, hypertension, coronary artery diseases and atherosclerosis (1). CVD is the leading cause of death worldwide among the aging population, and according to a previous study, CVD caused nearly 17.9 million deaths around the world (2). The main characteristic of CVD is atherosclerosis, which is considered closely related to the concentration of lipid storage and low-grade inflammation in the vascular wall caused by endothelial injury induced by accumulation of T cells and macrophages (3). Oxidation of lipids further leads to the accumulation of reactive oxygen species in the vascular wall, induce damage in cellular components and inflammation via promoting the activation of pro-atherogenic transcriptional factors (4). Taxifolin regulates various pharmacological processes, including anti-oxidation, mitochondrial protection and advanced glycation end products (AGE) formation suppression. It has become a potential treatment for cardiovascular diseases, neurocognitive disorders and malignancies (5). Taxifolin has presented an anti-inflammatory function reported by several researchers according to the previous study (6). A recent study found that taxifolin regulates the activation of NF-κB in a model of cerebral ischemia-reperfusion injury, which decreased the rate of infarction by 42% and 62% after middle cerebral arterial occlusion. Besides, taxifolin also inhibits the expression of COX-2 and iNOS and the expression of MAC-1 and ICAM-1 after cerebral ischemic reperfusion injury (7). Another study noticed that taxifolin suppressed the activation of NF-κB via the C-Fos signaling pathway using *in-vitro* and *in-vivo* experiments (8). Besides, taxifolin also suppressed the expression of IL-6, IL-1β and TNF-α (9) Thus we believed that taxifolin contributes to the treatment of cardiovascular disease through an anti-inflammatory effect. HMGB1 is firstly discovered in 1999 as a critical molecular in the inflammatory and infection process. A previous study found that after LPS stimulation, the expression of HMGB1 is increased in 16-32 h (10). Systemic HMGB1 levels *in-vivo* in mice started by 8 h and increased substantially from 16 to 32 h after LPS administration (2). Monocytes under HGMB1 stimulation would induce the release of multiple pro-inflammatory cytokines, including tumor necrosis factor (TNF), macrophage inflammatory protein-1 (MIP-1) and interleukins (ILs) (11). Treatment with monoclonal anti-HMGB1 antibodies would ameliorate tissue injury in the sepsis model (12). The recent study also confirmed that knockdown of HMGB1 using siRNA in macrophages and dendritic cells would reduce the secretion of cytokines (13). However, the detailed mechanism of HMGB1 in CVD remains unclear. A recent study noticed that taxifolin presented multiple functions that contribute to the potential novel therapeutic effect (14). Thus, we thought that inhibition of HMGB1 might contribute *in-vivo in-vitro* to the therapeutic effect of taxifolin in cardiovascular disease, and the present study also demonstrates the possible mechanism underlying these effects using *in-vivo* and *in-vitro* experiments using multiple methods might be a novel therapeutic strategy for cardiovascular disease. 

## Experimental

*Material and Methods*

Material. H-DMEM (10564011) and FBS (10100) were purchased from Gibco. Taxifolin (03890585) was purchased from Sigma (Darmstadt, Germany). Esp3I (ER0452), Lipofectamine 3000 Transfection Reagent (L3000015), TRIzol Plus RNA Purification Kit (12183555), High Capacity cDNA Reverse Transcription Kits (4368813) and TaqPath qPCR Master Mix (100020170) were purchased from Thermo (MA, USA). H9c2 cells (GNR5) and 293T cells (SCSP-502) were purchased from the National Collection of Authenticated Cell Culture (Shanghai, China). G418 (G8160) and puromycin (P8230) were purchased from Solarbio (Beijing, China). T4 PNK (M0201S) and Quick Ligase (M2200) were purchased from NEB (NY, USA). Anti- High mobility group box protein 1 (HMGB1, ab79823), hypoxia-inducible factor-1α (HIF-1α, ab1), vascular endothelial growth factor-α (VEGF-α, ab52917), transforming growth factor (TGF-β, ab92486), protein kinase B (AKT, ab179463), p-AKT (ab38449), mammalian target of rapamycin (mTOR, ab2732), p-mTOR (ab109268), signal transducer and activator of transcription 3 (STAT3, ab119352), p-STAT3 (ab76315), endothelial nitric oxide synthase (eNOS, ab76198), Fibroblast growth factor 21 (FGF, (ab171941) and Angiotensin-Converting Enzyme 1 (ACE1, ab75762) were purchased from Abcam. FGF21 (ab222506), VEGF-α (ab119566), TGF-β (ab100647), HIF-1α (ab171577) ELISA kits were purchased from Abcam (Cambridge, UK). 

Construct of vectors. Full-length of *HMGB1* cDNA was cloned via PCR using following primer: Forward: 

5'-CGGAATTCTTATGGGCAAAGGAGATCCTAAG-3'

Reverse:5’-ACAGATCTTTAGTGATGGTGATGGTGATGTTCATCATCATCATCTTC-3’. Production of *HMGB1* was digested with XhoI and BamHI and cloned into pCDNA3.1 vector to construct pCDNA3.1- *HMGB1* overexpression vector. Then, the vector was transfected into H9c2 cells using lipo 3000 transfection reagent for 48 h. The stably expressed cells were screened using 1000 μg/mL G418. *HMGB1* knockdown vector was constructed using the CRISPR/Cas9 system (15). Briefly, oligos were obtained using the following primer: Forward: 5’-CAGATACTCACGGAGGCCTCT-3’, Reverse:

5’-CAGAGGCCTCCGTGAGTATCTGCAAA-3’, and phosphorylated using T4 PNK. CRISPR plasmid was digested using BsmBI at 37 °C for 30 min. Plasmid and oligos were ligated using Quick Ligase, and *HGMB1* knockdown lentiviral vector was obtained after transferred into 293T cells. The stable *HGMB1* knockdown cells were screened using 2 μg/mL puromycin.

Cell culture and grouping. Cells were divided into four groups, including control group (NC), 100 μg/mL taxifolin treatment group (TG), *HMGB1* knockdown combined with 100 μg/mL taxifolin treatment group (KT) and *HMGB1* overexpression combined with 100 μg/mL taxifolin treatment group (OT) (16). Cells were cultured at a humidity 37 °C atmosphere, supplied with 5% CO_2_ using H-DMEM medium with 10% FBS. 

Ethical statement. The experiments performed according to the ethics committee of the Fourth Center Hospital of Tianjin (Tianjin, China) and the treatment of all 20 animals complied with the Public Health Service Policy on the Humane Care and Use of Laboratory Animals of the National Institutes of Health (SCXK 2019-0002).

Mice model construction. *HMGB1* knockdown (n = 5) and *HMGB1* overexpression (n = 5) mice were obtained from Cyagen Biotechnology Company (Suzhou, China). Normal mice, *HMGB1* knockdown and overexpression mice were kept in a 22 ± 1 °C (relative humidity: 55% ± 5%; light and dark cycle: 12 h light/12 h dark) atmosphere with water and food freely available. Mice were divided into four groups: normal control (NC), 200 mg/kg taxifolin treatment group (TG), *HMGB1 *knockdown combined with 200 mg/kg taxifolin treatment group (KT) and *HMGB1* overexpression combined with 200 mg/kg taxifolin treatment group (OT) (17). Mice were treated with taxifolin for 7 continuous days. After treatment, aorta tissue and serum sample in each group were collected to perform the following experiments. 

MTT assay. Cells were seeded into each well of a 96-well plate and cultured for 24 h. Then cells were treated with 100 μg/mL taxifolin for 24 h, followed by incubation with 5 mg/mL MTT for 4 h after being washed with sterile PBS three times. The OD value was measured at 490 nm using a microplate reader (Multiskan, Thermo). 

RNA extraction. RNA extraction was performed according to the protocol. Briefly, cells and aorta tissue were seeded into a 100 mm plate and cultured until the confluence reached 85-90%. Then, cells were lysed with TRIzol lysis buffer and incubated at room temperature for 5 min, followed by incubation with chloroform. The supernatant was collected after centrifuging at 12000×*g* for 15 min. The aqueous phase was collected into a new tube and then mixed with an equal amount of 70% ethanol. Then samples were loaded onto a spin cartridge with a collection tube and centrifuge at 12000×g for 15 s until all of the samples ﬂow through the tube. After a wash with wash buffer I and II, RNA was eluted with RNase-free water and stored at -80 °C until performing the following experiments. 

Reverse transcription. The reverse transcription was performed according to the protocol. The reaction mixture was made up as recommended, and the reaction was performed at 25 °C for 10 min, 37 °C for 120 min and 85 °C for 5 min. The production was stored at -80 °C until performing the following experiments.

Real-time quantitative polymerase chain reaction (qPCR). qPCR was performed according to the protocol. Primers used in qPCR were listed as follows: 

*HIF-1α*: Forward: 5’-GGGGAGGACGATGAACATCAA-3’; Reverse: 5’-GGGTGGTTTCTTGTACCCACA-3’;

*VEGF-α*: Forward:5’-TGTGAATGCAGACCAAAGAAAGA-3’; Reverse: ’-CACCAACGTACACGCTCCAG-3’;

*FGF21*: Forward:5’-GGAATTCAGATCTATGCACCACCACCACCACCACCACCCCATCCCTGACTCCAG-3’; Reverse:5’-GGCGATATCGCGGCCGCTCTAGATCAGGAAGCGTAGCTGGG-3’; 

*TGF-β*: Forward: 5’-TTTTTGTGGAGTAGTTAGATAGT-3’; Reverse: 

5’-AACTACTCCTCAACAACTCCTT-3’. Briefly, the reaction mixture was made up as recommended, and the reaction steps were set as follows: 50 °C for 2 min, 95 °C for 20 s and these steps were repeated for 40 times: 95 °C for 15 s, 60 °C for 1 min. The expression of each gene was calculated using 2^-ΔΔCt ^method. GAPDH was used as an internal control, and each experiment was repeated three times.

Protein extraction and Western blotting analysis. Cells and aorta tissue were grouped and treated as previously described; the cells were lysed with SDS lysis buffer (P0013G, Beyotime) on ice for 30 min. After centrifuged at 12000 rpm for 10 min, the supernatant was collected, and protein samples were determined using BCA assay. 60 μg protein samples were used to perform Western blotting analysis. Protein samples were loaded and separated using 10% SDS-PAGE, and after electrophoresis, proteins were transferred onto nitrocellulose membrane using Trans-Blot Turbo transfer system. Then, membranes were first incubated with 5% skim milk for 1 h at room temperature followed by incubation with primary antibodies overnight at 4 °C. The expression of each protein was detected using chemiluminescence after incubated with a secondary antibody for 1 h at room temperature. The gray value of each protein was calculated using Image Pro Plus 6.0 (MD, USA) and normalized with GAPDH. Each experiment was repeated three times independently. 

Enzyme-linked immunosorbent assay (ELISA). ELISA was performed according to the protocol of ELISA kits. Briefly, 100 μL of cultured medium and serum sample were added into each well of a 96-well plate and incubated at 4 °C overnight. After washed with wash buffer, samples were incubated with HRP-Streptavidin solution for 45 min at room temperature, followed with incubation with TMB substrate reagent for 30 min away from light. The OD value at 450 nm was measured using a microplate reader (Multiskan, Thermo) after the stop solution was added. 

Statistical analysis. Data were presented as mean ± SD. Differences between groups were calculated using one-way ANOVA analysis. *P < *0.05 was set as a statistical difference. Each experiment was repeated three times independently. 

## Results

**Figure 1:**
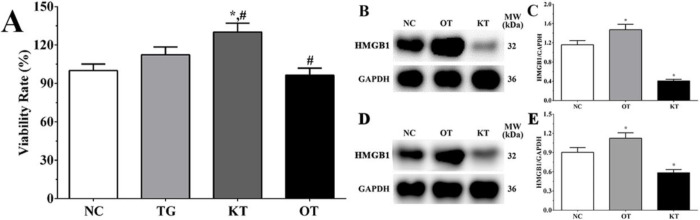
Effect of taxifolin on viability rate of H9c2 cells in each group. (A) Viability rate of H9c2 cells in each group. (B) Detection of HMGB1 in each group of cardiomyocytes. (C) Quantitative analysis of HMGB1 expression. (D) Detection of HMGB1 in each group of mice model. (E) Quantitative analysis of HMGB1 expression. ^*^P < 0.05 *vs. *NC group, ^#^*P < *0.05 *vs. *TG group. Data was presented as a mean ± SD. Each experiment was repeated for three times independently

Detection of the expression of HMGB1 in each group. As shown in Figure 1B, the expression of HMGB1 in NC, OT and KT group of cardiomyocytes were 1.16 ± 0.09, 1.47 ± 0.11, 0.41 ± 0.03. And compared with the NC group, the expression of HMGB1 in the OT group was significantly increased (*P < *0.05) while the expression of HMGB1 in the KT group was significantly decreased (*P < *0.05). And as shown in Figure 1D, the expression of HMGB1 in the NC, OT and KT group of mice were 0.90 ± 0.08, 1.08 ± 0.09 and 0.59 ± 0.05. Compared with the NC group, the expression of HMGB1 in the OT group was significantly increased (*P < *0.05) and the expression of HMGB1 was significantly decreased in the KT group (*P < *0.05). This result is indicating that the cell model used in this experiment was successfully established. 

Effect of taxifolin on the proliferation of H9c2 cells. The viability rate in TG, KT and OT groups were 112.6 ± 8.4, 126.3 ± 9.6, 92.1 ± 7.2%. Compared with the NC group, the viability rate in the KT group was significantly increased (*P < *0.05). And compared with the TG group, the viability rate was significantly increased in the KT group (*P < *0.05) and significantly decreased in the OT group (*P < *0.05). 

**Figure 2:**
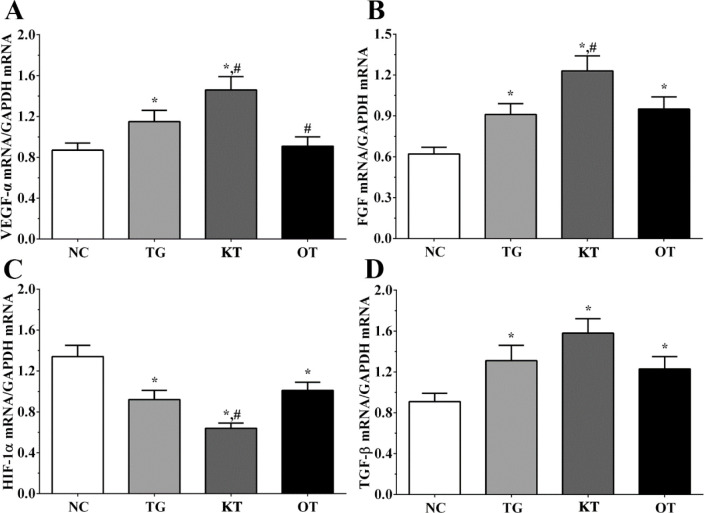
Effect of taxifolin on expression of angiogenesis related genes of cardiomyocytes. (A) Expression of VEGF-α mRNA. (B) Expression of FGF mRNA. (C) Expression of HIF-1α mRNA. (D) Expression of TGF-β mRNA. ^*^P < 0.05 *vs. *NC group, ^#^*P < *0.05 *vs. *TG group. Data was presented as a mean ± SD. Each experiment was repeated for three times independently

Effect of taxifolin on the expression of angiogenesis-related genes in cardiomyocytes. As shown in Figure 2, the expression of *FGF* in NC, TG, KT and OT groups were 0.62 ± 0.05, 0.92 ± 0.08, 1.22 ± 0.14, 0.93 ± 0.11. The expression of *FGF* was significantly increased in all treatment groups compared with the NC group (*P < *0.05). It was significantly increased in the KT group (*P < *0.05) compared with the TG group. The expression of *HIF-1α* in these groups were 1.36 ± 0.11, 0.88 ± 0.06, 0.62 ± 0.04, 0.97 ± 0.08. The expression of *HIF-1α* was significantly decreased in all treatment groups compared with the NC group (*P < *0.05), and compared with the TG group, the expression of *HIF-1α* in the KT group was significantly decreased (*P < *0.05). The expression of *TGF-β* in these groups were 0.91 ± 0.06, 1.24 ± 0.13, 1.52 ± 0.11, 1.21 ± 0.09. The expression of *TGF-β* was significantly increased in all treatment groups compared with the NC group. The expression of *VEGF-α* in these groups were 0.84 ± 0.04, 1.15 ± 0.11, 1.42 ± 0.13, 0.88 ± 0.05. Compared with the NC group, the expression of *VEGF-α* was significantly increased in TG and KT group (*P < *0.05) and was significantly increased in the KT group (*P < *0.05) and significantly decreased in the OT group (*P < *0.05) compared with TG group. 

**Figure 3:**
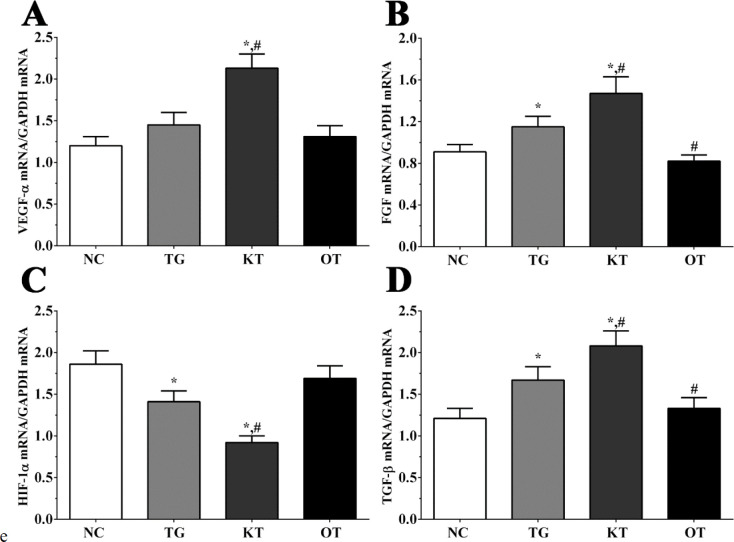
Effect of taxifolin on expression of angiogenesis related genes of mice model. (A) Expression of VEGF-α mRNA. (B) Expression of FGF mRNA. (C) Expression of HIF-1α mRNA. (D) Expression of TGF-β mRNA. ^*^P < 0.05 *vs. *NC group, ^#^*P < *0.05 *vs. *TG group. Data was presented as a mean ± SD. Each experiment was repeated for three times independently

Effect of taxifolin on the expression of angiogenesis-related genes in mice model. As shown in Figure 3, the expression of *FGF* in NC, TG, KT and OT groups were 0.91 ± 0.07, 1.15 ± 0.10, 1.47 ± 0.16, 0.82 ± 0.06. The expression of *FGF* was significantly increased in TG and KT groups compared with the NC group (*P < *0.05) and was significantly increased in the KT group (*P < *0.05) and significantly decreased in the OT group (*P < *0.05) compared with TG group. The expression of *HIF-1α* in these groups were 1.86 ± 0.16, 1.41 ± 0.13, 0.92 ± 0.08, 1.69 ± 0.15. The expression of *HIF-1α* was significantly decreased in TG and KT groups compared with the NC group (*P < *0.05), and compared with the TG group, the expression of HIF-1α in the KT group was significantly decreased (*P < *0.05). The expression of *TGF-β* in these groups were 1.21 ± 0.12, 1.67 ± 0.16, 2.08 ± 0.18, 1.33 ± 0.13. The expression of *TGF-β* was significantly increased in TG and KT groups compared with the NC group (*P < *0.05) and significantly increased in the KT group (*P < *0.05) and significantly decreased in the OT group (*P < *0.05) compared with the TG group. The expression of *VEGF-α* in these groups were 1.20 ± 0.11, 1.45 ± 0.15, 2.13 ± 0.17, 1.31 ± 0.13. Compared with NC and TG groups, the expression of *VEGF-α* was significantly increased in the KT group (*P < *0.05).

**Figure 4:**
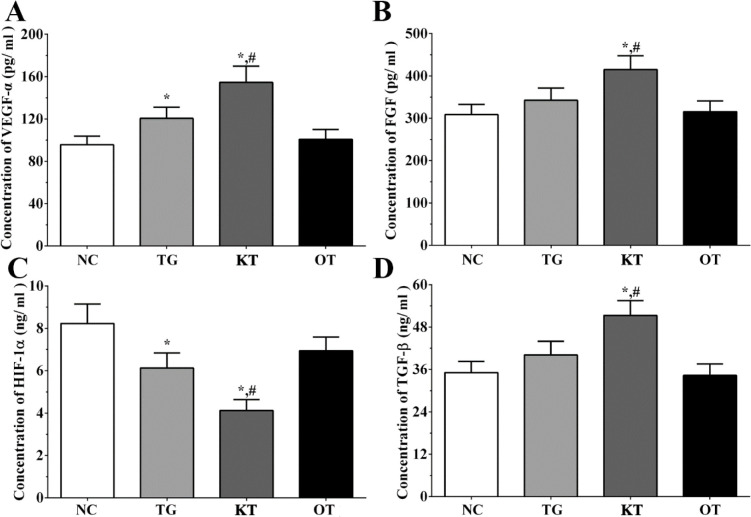
Effect of taxifolin on expression of angiogenesis related factors in cultured medium of H9c2 cells. (A) Concentration of VEGF-α. (B) Concentration of FGF. (C) Concentration of HIF-1α. (D) Concentration of TGF-β. ^*^P < 0.05 *vs. *NC group, ^#^*P < *0.05 *vs. *TG group. Data was presented as a mean ± SD. Each experiment was repeated for three times independently

Effect of taxifolin on the secretion of angiogenesis-related genes in the cultured medium of H9c2 cells. As shown in Figure 4, the concentration of FGF in the cultured medium of H9c2 cells in NC, TG, KT and OT groups were 306.2 ± 15.3, 335.1 ± 18.2, 415.3 ± 23.1, 303.2 ± 14.6 pg/mL. The concentration of FGF was significantly increased in the KT group (*P < *0.05) compared with NC and TG groups. The concentration of HIF-1α in cultured medium of H9c2 cells in each group were 8.2 ± 0.9, 6.1 ± 0.8, 3.9 ± 0.4, 6.7 ± 0.6 ng/mL. The concentration of HIF-1α was significantly decreased in TG and KT group (*P < *0.05) compared with the NC group and was significantly decreased in the KT group (*P < *0.05) compared with the TG group. The concentration of TGF-β in the cultured medium of H9c2 cells in each group were 35.6 ± 3.6, 38.8 ± 4.3, 50.6 ± 5.2, 32.6 ± 3.5 ng/mL. The concentration of TGF-β was significantly increased in the KT group compared with NC and TG group. The concentration of VEGF-α in the cultured medium of H9c2 cells in each group were 94.1 ± 9.8, 118.2 ± 11.3, 149.6 ± 18.4, 97.5 ± 10.2 pg/mL. The concentration of VEGF-α was significantly increased in TG and KT group (*P < *0.05) compared with the NC group and was significantly increased in the KT group (*P < *0.05) compared with the TG group. 

**Figure 5:**
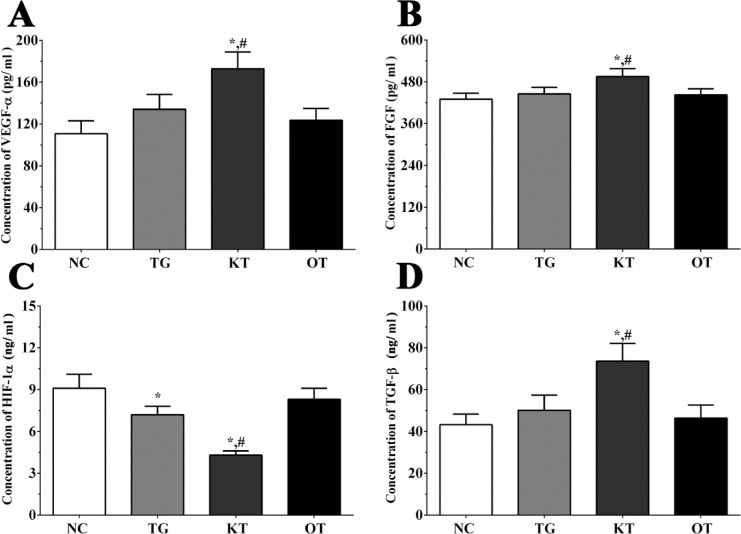
Effect of taxifolin on expression of angiogenesis related factors in serum sample of mice model. (A) Concentration of VEGF-α. (B) Concentration of FGF. (C) Concentration of HIF-1α. (D) Concentration of TGF-β. ^*^P < 0.05 *vs. *NC group, ^#^*P < *0.05 *vs. *TG group. Data was presented as a mean ± SD. Each experiment was repeated for three times independently

Effect of taxifolin on the secretion of angiogenesis-related genes in serum sample of mice. As shown in Figure 5, the concentration of FGF in serum samples of mice in NC, TG, KT and OT groups were 430.2 ± 17.3, 445.2 ± 19.1, 495.3 ± 22.4, 442.4 ± 17.7 pg/mL. The concentration of FGF was significantly increased in the KT group (*P < *0.05) compared with NC and TG group. The concentration of HIF-1α in each group were 9.1 ± 1.0, 7.2 ± 0.6, 4.3 ± 0.3, 8.3 ± 0.8 ng/mL. The concentration of HIF-1α was significantly decreased in TG and KT group (*P < *0.05) compared with the NC group and was significantly decreased in the KT group (*P < *0.05) compared with the TG group. The concentration of TGF-β in each group were 43.2 ± 5.1, 50.1 ± 7.3, 73.6 ± 8.5, 46.4 ± 6.2 ng/mL. The concentration of TGF-β was significantly increased in the KT group compared with NC and TG group. The concentration of VEGF-α in each group were 110.6 ± 12.4, 134.1 ± 14.1, 172.8 ± 16.1, 123.5 ± 11.4 pg/mL. The concentration of VEGF-α was significantly increased in the KT group (*P < *0.05) compared with the NC group and was significantly increased in the KT group (*P < *0.05) compared with the TG group. 

**Figure 6:**
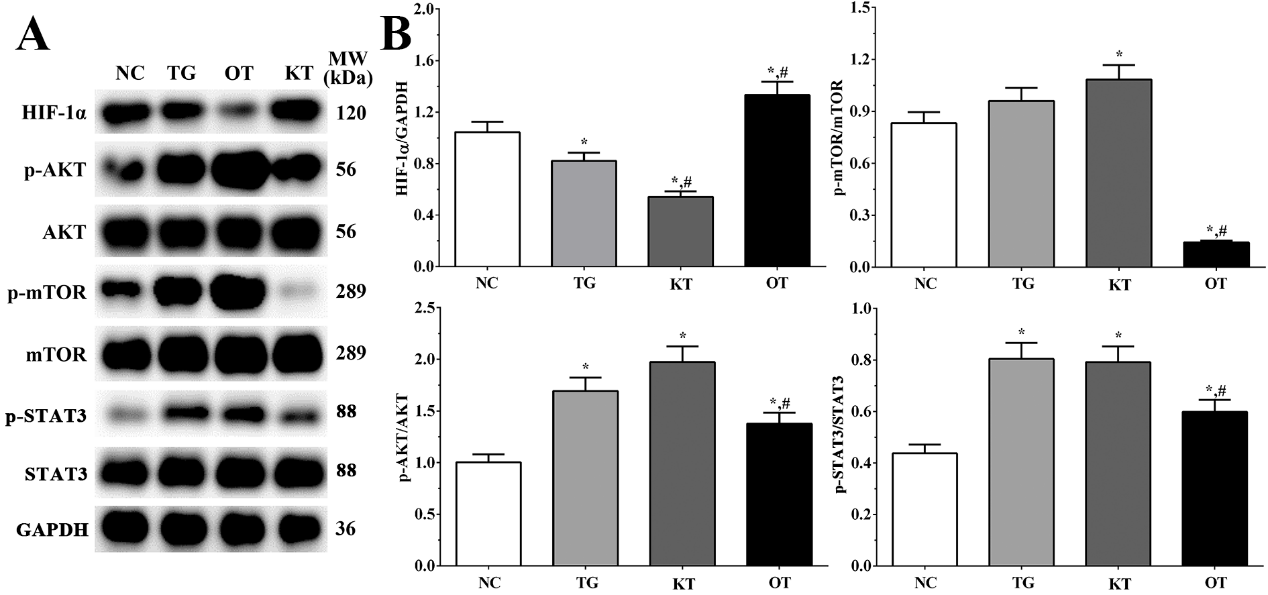
Effect of taxifolin on activation of PI3K/AKT/mTOR/STAT3 signaling pathway of cardiomyocytes. (A) Western blotting analysis of PI3K/AKT/mTOR/STAT3 signaling pathway. (B) Quantitative analysis of PI3K/AKT/mTOR/STAT3 signaling pathway. ^*^P < 0.05 *vs. *NC group, ^#^*P < *0.05 *vs. *TG group. Data was presented as a mean ± SD. Each experiment was repeated for three times independently

Effect of taxifolin on activation of PI3K/AKT/mTOR/STAT3 signaling pathway in cardiomyocytes. As shown in Figure 6, the activation of the PI3K/AKT/mTOR/STAT3 signaling pathway was determined using Western blotting analysis. The ratio of p-AKT/AKT in NC, TG, KT and OT groups were 1.00 ± 0.08, 1.69 ± 0.13, 1.97 ± 0.15 and 1.38 ± 0.11. The ratio of p-AKT/AKT was significantly increased in all treatment groups compared with the NC group (*P < *0.05), and the ratio of p-AKT/AKT was significantly decreased in the OT group compared with the TG group (*P < *0.05). The ratio of p-mTOR/mTOR in these groups were 0.83 ± 0.06, 0.96 ± 0.07, 1.08 ± 0.08 and 0.14 ± 0.01. The ratio of p-mTOR/mTOR was significantly increased in the KT group (*P < *0.05) and significantly decreased in the OT group (*P < *0.05) compared with the NC group and was significantly decreased in the OT group (*P < *0.05) compared with TG group. The ratio of p-STAT3/STAT3 were 0.44 ± 0.03, 0.80 ± 0.06, 0.79 ± 0.06 and 0.60 ± 0.05. The ratio of p-STAT3/STAT3 was significantly increased in all treatment groups (*P < *0.05) compared with the NC group and was significantly decreased in the OT group (*P < *0.05) compared with the TG group. The expression of HIF-1α in each group was 1.24 ± 0.10, 1.03 ± 0.09, 0.60 ± 0.05 and 1.35 ± 0.11. The expression was significantly decreased in TG and KT group (*P < *0.05) and significantly increased in the OT group (*P < *0.05) compared with the NC group, the expression was significantly decreased in the KT group (*P < *0.05) and significantly increased in OT group (*P < *0.05) compared with TG group. 

**Figure 7:**
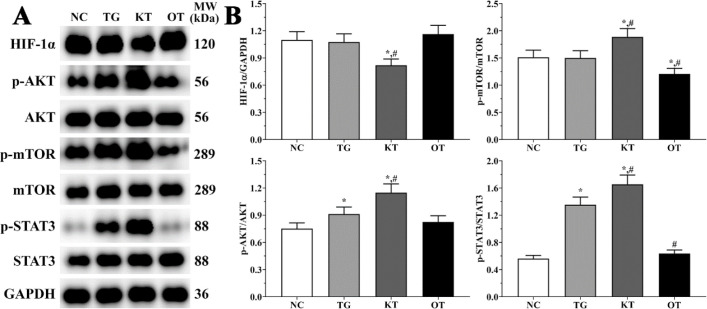
Effect of taxifolin on activation of PI3K/AKT/mTOR/STAT3 signaling pathway of mice model. (A) Western blotting analysis of PI3K/AKT/mTOR/STAT3 signaling pathway. (B) Quantitative analysis of PI3K/AKT/mTOR/STAT3 signaling pathway. ^*^P < 0.05 *vs. *NC group, ^#^*P < *0.05 *vs. *TG group. Data was presented as a mean ± SD. Each experiment was repeated for three times independently

Effect of taxifolin on activation of PI3K/AKT/mTOR/STAT3 signaling pathway in mice model. As shown in Figure 7, the activation of the PI3K/AKT/mTOR/STAT3 signaling pathway was determined using Western blotting analysis. The ratio of p-AKT/AKT in NC, TG, KT and OT groups were 0.75 ± 0.06, 0.91 ± 0.08, 1.15 ± 0.10 and 0.82 ± 0.07. The ratio of p-AKT/AKT was significantly increased in TG and KT groups compared with the NC group (*P < *0.05), and the ratio of p-AKT/AKT was significantly increased in the KT group compared with the TG group (*P < *0.05). The ratio of p-mTOR/mTOR in these groups were 1.51 ± 0.13, 1.50 ± 0.13, 1.89 ± 0.16 and 1.21 ± 0.10. The ratio of p-mTOR/mTOR was significantly increased in the KT group (*P < *0.05) and significantly decreased in the OT group (*P < *0.05) compared with NC and TG group. The ratio of p-STAT3/STAT3 were 0.56 ± 0.05, 1.35 ± 0.11, 1.65 ± 0.14 and 0.64 ± 0.05. The ratio of p-STAT3/STAT3 was significantly increased in TG and KT groups (*P < *0.05) compared with the NC group and was significantly increased in the KT group (*P < *0.05) and significantly decreased in the OT group (*P < *0.05) compared with TG group. The expression of HIF-1α in these group were 1.10 ± 0.09, 1.08 ± 0.09, 0.82 ± 0.07 and 1.16 ± 0.10. The expression of HIF-1α was significantly decreased in the KT group (*P < *0.05) compared with NC and TG group.

**Figure 8:**
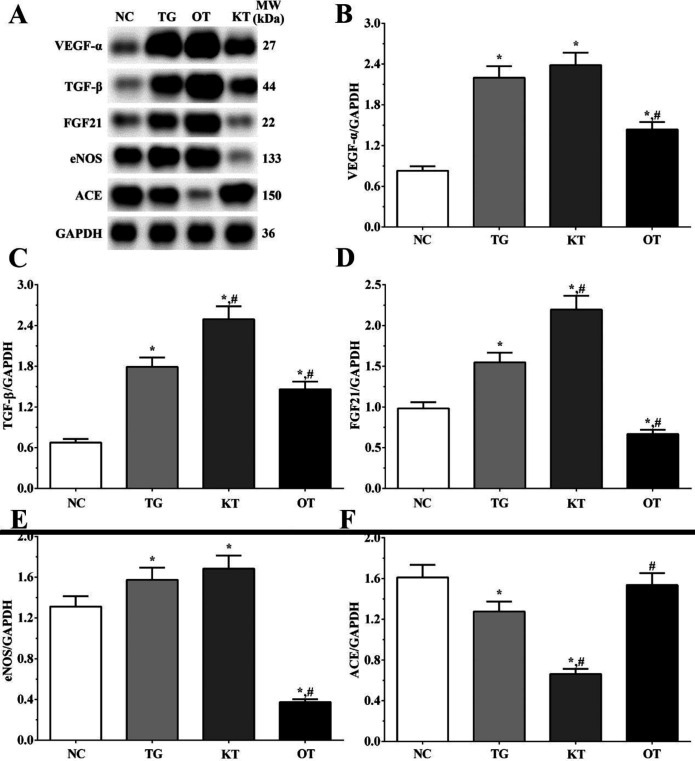
Effect of taxifolin on expression of angiogenesis related proteins of cardiomyocytes. (A) Western blotting analysis of angiogenesis related proteins. (B, C, D, E and F) Quantitative analysis of angiogenesis related proteins. ^*^P < 0.05 *vs. *NC group, ^#^*P < *0.05 *vs. *TG group. Data was presented as a mean ± SD. Each experiment was repeated for three times independently

Effect of taxifolin on expression angiogenesis-related proteins in H9c2 cells. As shown in Figure 8, the expression of angiogenesis-related proteins was determined using Western blotting analysis. The expression of VEGF-α were 0.83 ± 0.06, 2.20 ± 0.17, 2.38 ± 0.18 and 1.44 ± 0.11 in NC, TG, KT and OT groups. The expression of VEGF-α was significantly increased in all treatment groups (*P < *0.05) compared with the NC group and was significantly decreased in the OT group (*P < *0.05) compared with the TG group. The expression of TGF-β were 0.68 ± 0.05, 1.79 ± 0.14, 2.49 ± 0.19 and 1.46 ± 0.11 in these groups. The expression of TGF-β aswas significantly increased in all treatment groups compared with the NC group (*P < *0.05) and was significantly increased in the KT group (*P < *0.05) and significantly decreased in the OT group (*P < *0.05) compared with TG group. The expression of FGF21 in these groups were 0.98 ± 0.08, 1.55 ± 0.12, 2.20 ± 0.17 and 0.67 ± 0.05 in these groups. The expression of FGF21 was significantly increased in TG and KT groups compared with the NC group (*P < *0.05) and was significantly decreased in the OT group (*P < *0.05). The expression of FGF21 was significantly increased in the KT group (*P < *0.05) and was significantly decreased in the OT group (*P < *0.05) compared with the TG group. The expression of eNOS were 1.31 ± 0.10, 1.57 ± 0.12, 1.68 ± 0.13 and 0.37 ± 0.03. The expression of eNOS was significantly increased in the TG and KT groups (*P < *0.05) and was significantly decreased in the OT group (*P < *0.05) compared with the NC group. And the expression of eNOS was significantly decreased in the OT group (*P < *0.05) compared with the TG group. The expression of ACE in these groups were 1.61 ± 0.12, 1.28 ± 0.10, 0.66 ± 0.05 and 1.54 ± 0.12. The expression of ACE was significantly decreased in TG and KT groups (*P < *0.05) compared with the NC group, and compared with the TG group, the expression of ACE was significantly decreased in the KT group (*P < *0.05) and significantly increased in OT group (*P < *0.05) compared with TG group. 

**Figure 9:**
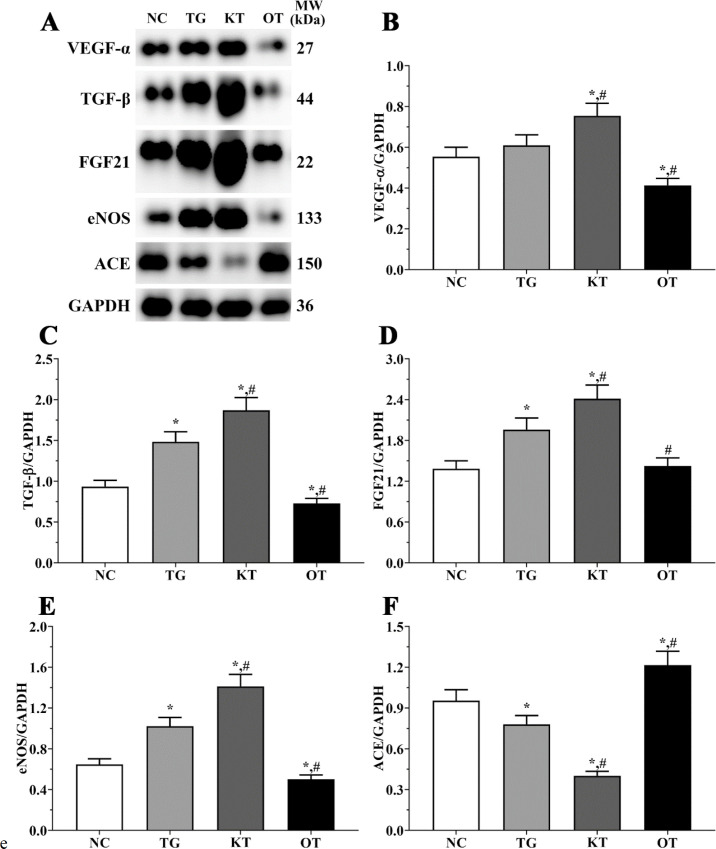
Effect of taxifolin on expression of angiogenesis related proteins of mice model. (A) Western blotting analysis of angiogenesis related proteins. (B, C, D, E and F) Quantitative analysis of angiogenesis related proteins. ^*^P < 0.05 *vs. *NC group, ^#^*P < *0.05 *vs. *TG group. Data was presented as a mean ± SD. Each experiment was repeated for three times independently

Effect of taxifolin on expression angiogenesis-related proteins in mice model. As shown in Figure 9, the expression of angiogenesis-related proteins was determined using Western blotting analysis. The expression of VEGF-α were 0.55 ± 0.05, 0.61 ± 0.05, 0.75 ± 0.06 and 0.41 ± 0.03 in NC, TG, KT and OT groups. The expression of VEGF-α was significantly increased in the KT group (*P < *0.05) and significantly decreased in the OT group (*P < *0.05) compared with NC and TG group. The expression of TGF-β were 0.93 ± 0.08, 1.48 ± 0.12, 1.87 ± 0.16 and 0.73 ± 0.06 in these groups. The expression of TGF-β were significantly increased in TG and KT groups compared with the NC group (*P < *0.05) and significantly decreased in the OT group (*P < *0.05) compared with the NC group, and was significantly increased in the KT group (*P < *0.05) and significantly decreased in OT group (*P < *0.05) compared with TG group. The expression of FGF21 in these groups were 0.98 ± 0.08, 1.55 ± 0.12, 2.20 ± 0.17 and 0.67 ± 0.05 in these groups. The expression of FGF21 were significantly increased in TG and KT groups compared with the NC group (*P < *0.05). The expression of FGF21 was significantly increased in the KT group (*P < *0.05) and was significantly decreased in the OT group (*P < *0.05) compared with the TG group. The expression of eNOS were 0.65 ± 0.05, 1.02 ± 0.09, 1.41 ± 0.12 and 0.50 ± 0.04. The expression of eNOS was significantly increased in the TG and KT groups (*P < *0.05) and was significantly decreased in the OT group (*P < *0.05) compared with the NC group. And was significantly increased in the KT group (*P < *0.05) and significantly decreased in the OT group (*P < *0.05) compared with the TG group. The expression of ACE in these groups were 0.95 ± 0.08, 0.78 ± 0.06, 0.40 ± 0.03 and 1.22 ± 0.10. The expression of ACE was significantly decreased in the TG and KT groups (*P < *0.05) and significantly increased in the OT group (*P < *0.05) compared with the NC group, and compared with the TG group, the expression of ACE was significantly decreased in KT group (*P < *0.05) and significantly increased in OT group (*P < *0.05).

## Discussion

**Figure 10:**
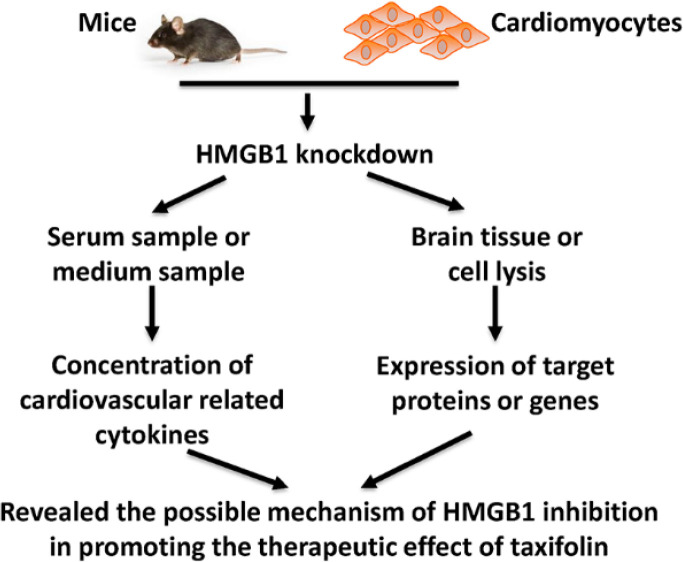
Schematic figure of present study

According to World Health Organization (WHO), cardiovascular diseases (CVD) are a severe disease of the cardiovascular system and caused 17 million people death worldwide each year according to World Health Organization (WHO). CVD affects patients’ quality of life and costs treatment of spending each year (18, 19). High mobility group box 1 (HMGB1) has been identified as a critical mediator of severe sepsis. Ketamine has been shown to reduce sepsis-induced pathological complications. These effects are because of the reduced expression and release of several inflammatory mediators. Taxifolin (3,5,7,3’,4’-pentahydroxy-flavanone) belongs to the flavonoid, obtained from citrus fruits, grapes, olive oil, and onions (20). A previous study found that taxifolin presented a wide range of biochemical and pharmacological effects, including anti-tumor, anti-inflammatory and systematic protective function (21, 22). In this study, as shown in Figure 10, we established HMGB1 overexpression and knockdown cell model in H9c2 cells and found that HMGB1 might enhance the protective role of taxifolin in H9c2 cells through PI3K/AKT/mTOR/ERK signaling pathway. 

Multiple extracellular stimuli could activate the PI3K/AKT signaling pathway, including fibroblast growth factor receptor (FGFR), typically epidermal growth factor receptor (EGFR) and insulin-like growth factor I receptor (IGF-IR). AKT in the central mediator of the PI3K/AKT signaling pathway, leading to the phosphorylation of multiple downstream molecules (23), participated in various physiological processes, such as cellular activation, inflammatory response and apoptosis (24). mTOR is a serine-threonine protein kinase, contains two complexes, mTORC1 and mTORC2, which performs an important role in regulating cellular growth and survival. AKT could be recruited at the plasma membrane and phosphorylated at S473 by mTORC2, which further promotes cellular proliferation. On the other hand, phosphorylated AKT promotes phosphorylation of multiple downstream molecules, such as Bcl-2 and mTOR, protect cells from apoptosis (25). A previous study found that activation of STAT3 via phosphorylation reduces ROS accumulation and cellular apoptosis with increasing angiogenesis. Knockout of STAT3 enlarged the infarct size followed IR (26). Besides, expression of STAT3 in cardiomyocytes contributes to the remodeling during MI (27), and inhibition of STAT3 expression increase the expression of pro-apoptotic molecules (28). Up-regulation in STAT3 expression promotes the expression of ROS scavengers and antioxidants (29), and on the other hand, STAT3 could reduce the generation of ROS by inhibition of complex I and III of electron transport chain (30). And in this study, taxifolin treatment would significantly activates PI3K/AKT/mTOR/STAT3 signaling pathway, presented a protective role. While knockdown of HMGB1 would enlarge this trend and overexpression of HMGB1 would reduce this trend, indicating that knockdown of HMGB1 might enhance the therapeutic effect of taxifolin on cardiomyocytess.

HIF-1α is a downstream molecular of the PI3K/AKT/mTOR signaling pathway, also plays an important role in response to hypoxia stress. Under normal status, HIF-1α was rapidly degraded while maintaining stability under hypoxia status (31). In response to hypoxia, the inflammatory response in cardiomyocytes was activated, resulting in releasing inflammatory cytokines and growth factors, promoting phagocyte adherence to the vascular wall. Main growth factors participate in this process were vascular endothelial growth factor-α (VEGF-α) and nitric oxide (NO). VEGF family was firstly identified from medium of tumor cell lines as a vascular leakiness molecular (32). VEGF-α is a member of VEGF family, which could bind with heparin and plays a critical role in angiogenesis (33). Previous study found that VEGF-α could promote the growth and migration of ECs, thus induce angiogenesis in various animal models (34). Besides, VEGF-α was found to mobilize endothelial progenitor cells (EPCs), further contributes to vasculogenesis and endothelialization of injured vessels (35). Nitric oxide (NO) derived from ECs could diastole blood vessels and inhibit adhesion and aggregation of platelet, and could also restrain the proliferation of smooth muscle cells (36). Under normal status, NO is mostly produced by ECs via oxidates guanidino nitrogen of l-Arg end by eNOS. NO is also an important signaling molecule inhibits vascular lesion formation process, as well as reduce the production of reactive oxygen species (ROS) and lipid peroxidation (37). 

An up-regulation of VEGF-α and NO could promote the proliferation of cardiomyocytes, as well as angiogenesis function (38). Fibroblast growth factors (FGFs) are a family of growth factors expressed in various species and involved in various biological processes, including organogenesis, homeostasis and metabolism. Among them, FGF21 seems closely related to heart development, health and disease. A previous study found that cardiac hypertrophy was more severe in FGF21 knockout mice, while this trend could be reversed after supplied with FGF21 in both FGF21 knockout mice and cultured cells. Secretion of FGF21 could also inhibit the hypertrophic cardiomyopathycaused by isoproterenol (39). Transforming growth factor β (TGF-β) is a protein secreted by various cell types and plays an important role in regulating cellular function via cell-to-cell signaling. TGF-β could induce cellular proliferation, adhesion, extracellular matrix (ECM) protein production and cellular migration (40). TGF-β could stimulate the proliferation of smooth muscle cells (SMCs) by inducing growth factors. This effect of TGF-β is mediated by multiple signaling pathways, including p38 MAP kinase, ERK MAP kinases, and PI3K-AKT signaling pathways (41). Besides, TGF-β could bind with ALKs expressed at the surface of ECs, resulting in a new vessel, EC proliferation and migration (42). Angiotensin-converting enzyme (ACE) is an important enzyme in the renin-angiotensin system, regulating blood pressure. ACE converts AngI into AngII via removing His-Leu from C-terminal, and inhibition of ACE is one of the strategies for preventingcardiovascular disease, especially for hypertension (43). After treatment of taxifolin, the expression of HIF-1α was inhibited, indicating that taxifolin could increase the concentration of oxygen in H9c2 cells. Besides, the expressions of FGF21, VEGF-α, TGF-β and eNOS were also increased, indicating that taxifolin could promote the proliferation of ECs, as well as the production of NO, which performs a protective role. And knockdown of HMGB1 would enlarge the effect of taxifolin.

## Conclusion

We firstly established *HMGB1* overexpression and knockdown cell model found that taxifolin could decrease the expression of HIF-1α, while increase the expression of FGF21, VEGF-α, TGF-β and eNOS, thus increase the proliferation of ECs and production of NO, performs a protective role, resulting in increasing in viability rate of H9c2 cells. We also found that these effects might be mediated by activation of the PI3K/AKT/mTOR/STAT3 signaling pathway. And knockdown of *HMGB1* could enlarge the impact of taxifolin while overexpression of *HMGB1* would reduce it. Thus, we speculated that knockdown of *HMGB1* might be a new therapeutic strategy for knockdown of *HMGB1* could enhance the protective role of taxifolin in ECs. 
